# Prevalence of brucellosis and associated risk factors in dairy cattle in Maekel and Debub Regions, Eritrea

**DOI:** 10.3389/fvets.2023.1177572

**Published:** 2023-06-15

**Authors:** Ghebremeskel Habteyohannes Efrem, Bereket Mihreteab, Michael K. Ghebremariam, Tekeste Okbamichael, Yosief Ghebresilasie, Siobhan M. Mor, Gezahegne Mamo

**Affiliations:** ^1^National Animal and Plant Health, Laboratory, Ministry of Agriculture, Asmara, Eritrea; ^2^Department of Veterinary Microbiology, Immunology and Public Health, College of Veterinary Medicine and Agriculture, Addis Ababa University, Bishoftu, Ethiopia; ^3^Pathology Laboratory, National Animal and Plant Health Laboratory, Ministry of Agriculture, Asmara, Eritrea; ^4^Division of Pathology, Public Health & Disease Investigation, School of Biodiversity, One Health and Veterinary Medicine, University of Glasgow, Glasgow, United Kingdom; ^5^Serology Laboratory, National Animal and Plant Health Laboratory, Ministry of Agriculture, Asmara, Eritrea; ^6^Vaccine Production Unit, National Animal and Plant Health Laboratory, Ministry of Agriculture, Asmara, Eritrea; ^7^Institute of Infection, Veterinary and Ecological Sciences, University of Liverpool, Leahurst, Neston, United Kingdom; ^8^International Livestock Research Institution (ILRI), Nairobi, Ethiopia

**Keywords:** brucellosis, dairy cattle, prevalence, risk factor, Eritrea

## Abstract

**Introduction:**

Brucellosis is a zoonotic disease with worldwide distribution. It is considered endemic in Eritrea, however, the current prevalence status and related risk factors in animals are unknown. The objective of this study was to determine the prevalence of and risk factors for brucellosis in dairy cattle in Maekel and Debub regions, Eritrea.

**Methods:**

A cross sectional study was conducted between August 2021 and February 2022. A total of 2,740 dairy cattle from 214 herds in 10 sub-regions of Eritrea were selected for blood and data collection. Blood samples were tested using Rose Bengal Plate Test (RBPT) and positive samples were confirmed using competitive (c-ELISA). Data on risk factors was collected using questionnaire and analyzed using logistic regression.

**Results:**

In total, 34/2740 animals tested positive by RBPT. Of these, 29 were confirmed positive by c-ELISA, giving an apparent and estimated true individual-level prevalence of 1.1% (95% CI: 0.7, 1.5%) and 1.3% (95% CI: 0.9, 1.8%), respectively. Sixteen herds (7.5%) tested positive by RBPT and of these 15 herds (7.0%) were confirmed positive by c-ELISA, giving an estimated true herd-level prevalence of 7.0% (95% CI: 4.0, 10.7). Animal and herd-level apparent prevalence was 1.6 and 9.2% in Maekel, while in Debub it was 0.6 and 5.5%, respectively. Multivariable regression analysis indicated that non-pregnant lactating cows (adjusted odds ratio [aOR] = 3.35; *p* = 0.042) were more likely to be *Brucella* sero-positive. History of abortion on the farm (aOR = 5.71; *p* = 0.026) and larger number of cows in the herd (aOR = 1.14; *p* < 0.001) were associated with brucellosis sero-positivity in herds.

**Conclusion:**

Brucellosis prevalence was low in the study areas. Nonetheless, this low prevalence may increase if the disease is not controlled. Therefore, testing animals before movement, good farming practices, sanitary measures, and an awareness raising program on brucellosis are recommended.

## 1. Introduction

Worldwide, brucellosis is one of the most important zoonotic diseases with major implications for animal and human health (1). Brucellosis is caused by bacteria of the genus *Brucella* spp. Twelve species have been recognized of which the most important are *B. abortus* (primary host: cattle), *B. melitensis* (sheep and goats) and *B. suis* (swine) (2). Transmission of *Brucella* spp. between animals occurs via ingestion of contaminated feeds and water, inhalation of aerosolized bacteria, mating and direct contact with infected placenta and uterine discharges (3, 4). Brucellosis contributes to significant economic losses to cattle farmers through causing abortion, stillbirths, birth of weak calves, calf death, prolonged inter-calving intervals, and infertility (5, 6). Further, up to 20% reduction in milk yield can occur in carrier animals (7).

The main risk factors for animal brucellosis can be categorized into animal-associated, management-related, and environmental factors. Animal factors include age, sex, parity, breed, history of retained placenta, abortion, and milking method (5, 8, 9). Animal management-related risk factors include type of production system, breeding practices, whether new arrivals are screened, herd size, herd density, vaccination, hygiene and disinfection practices, and level of farmer awareness about the disease (8, 10). Environmental risk factors include climate and weather (humidity, temperature, and sunlight), size of grazing pasture, cleaned and disinfected areas and proximity to wildlife (11). Bovine brucellosis has been eradicated in many developed countries but remains endemic in developing countries as a result of inadequate policies, limited resources, lack of awareness about the disease and/or ineffective control programs (12).

In Eritrea, there are limited published data on brucellosis, and most are outdated or limited in design. Brucellosis was detected for the first time in Eritrea in 1943 in Agordet Gash-Barka region (13, 14) where 15% of the cows had a positive titre for brucellosis. However, the original report did not mention the type of cattle, method of sampling, sample size or the test methods used. In 1986, Tekleghiorghis (15) (in his D.V.M. Thesis) reported a prevalence of 28.8% among 322 dairy animals in Asmara using Standard Agglutination Test (SAT) and Rose Bengal Plate Test (RBPT). In 2000, Omer et al. (16) assessed the prevalence of brucellosis in cattle, goats, sheep, camels, and horses in different husbandry systems, as well as in high-risk, occupationally exposed humans (17); *Brucella* antibody was detected in all species except horses, with 8.2 and 35.9% individual- and herd-level prevalence recorded in dairy cattle in Asmara city. More recently, Scacchia et al. (18) analyzed 15,049 serum samples collected as part of a census of all sexually mature dairy cattle in five of the six regions of the country; an overall sero-prevalence of 2.8% was reported with the highest rate found in Maekel (5.2%) followed by Debub (2.0%) and Gash Barka (1.7%). Further, Samson (19) recorded individual and herd level sero-prevalence of around 3 and 2%, respectively, in dairy farms in Berik sub-region of Maekel region. In addition, several clinical reports and laboratory diagnostic results have indicated the presence of brucellosis in different localities in the county (20, 21).

Certain control measures against brucellosis in dairy cattle were introduced following the release of a legal notice by the Regulatory Services Department, under the Ministry of Agriculture, which specified requirements and standards for milk and milk products processing plants in 2006 (22). Subsequently, a survey of brucellosis was conducted from 2008 to 2010 in all dairy farms in the country and it was decided that all sero-positive animals would be slaughtered. However, the implementation process and its sustainability was not strictly followed up. For the last 20 or more years there has been no vaccination against brucellosis in dairy cattle in the country.

The previous reports are fragmented and considered insufficient for making decisions on the design of feasible control strategies for brucellosis in Eritrea. Updated information from studies conducted using statistically representative data are needed. More than 51% of the country’s dairy farms are found in Maekel and Debub regions (23). They are the main sources of milk and dairy products for most consumers residing in the two densely populated regions. This study was therefore designed to generate robust estimates of the prevalence of brucellosis and associated risk factors in Maekel and Debub regions, Eritrea.

## 2. Materials and methods

### 2.1. Study design and area

This cross-sectional study was conducted in Maekel and Debub regions of Eritrea between August 2021 and February 2022. Administratively, Eritrea is divided into 6 regions namely Maekel, Debub, Anseba, Gash Barka, Northern Red Sea (NRS), and Southern Red Sea (SRS). Maekel region is located in the central part of the country with latitude 150 ° 34′ 36” North and longitude of 380 °41′ 36″East. It is divided into four sub-regions with 59 administrative areas and 89 villages. Its population size ranks third, next to Debub region. It has a total area of 1,300 km^2^. Debub region is located in the southern part of the country with latitude 14 °53′ 14” N and 38 °48′ 55″E longitude. It is divided into 12 sub-regions, 217 administrative areas and 886 villages. It has second highest population of all the six regions and has a land area of 80,000 km^2^ (24).

### 2.2. Sampling design and sample size

Dairy cattle farms managed under intensive and semi-intensive production systems in all sub-regions of Maekel and Debub regions were the target of the study. Dairy farms with more than four animals aged 6 months or older were eligible for inclusion. To construct the sampling frame, lists of dairy farms (including herd size) were obtained from the respective Regional Ministry of Agriculture Veterinary Offices. According to these lists, the estimated dairy cattle population in Maekel and Debub regions was 4,696 and 7,778, belonging to 1,118 and 1,511 herds, respectively. Of these, 245 and 406 farms in Maekel and Debub regions owned more than four animals, respectively, and were thus eligible for inclusion in the study.

An initial crude sample size of 170 herds (*n*_1_) was calculated using Epitools online software^1^ assuming 10% expected true prevalence with 95% confidence level, desired precision of 5%, and combined sensitivity and specificity for RBPT and c-ELISA of 83 and 100%, respectively. The combined sensitivity and specificity were calculated using the following formulas for testing in series (25):


$$\text{Se}\left(\text{serial}\right)={\text{Se}}_{1}\times {\text{Se}}_{2}$$

$$\text{Sp}\left(\text{serial}\right)=1-\left[\left(1-{\text{Sp}}_{1}\right)\times \left(1-{\text{Sp}}_{2}\right)\right]$$

where individual test sensitivity (Se_1_) and specificity (Sp_1_) of RBPT was 87 and 97.8%, respectively, and sensitivity (Se_2_) and specificity (Sp_2_) of c-ELISA was 95.2 and 99.7%, respectively (26). The crude sample size (*n*_1_) was subsequently adjusted for finite population using the following formula (27):


$$n_{\text{adj}}=1/\left(\frac{1}{n}+\frac{1}{N}\right)$$

where *n*_adj_ = adjusted sample size, *N* = finite population size (651 herds), *n* = sample size calculated for infinite population (170), giving an adjusted sample size (*n*_adj_) of 135. Finally, to increase precision of the estimate, a design effect of 1.56 was applied giving a minimum sample size of 210 herds (*n*_2_). This design effect was assumed considering the absence of any previous similar study conducted in the country that can be used to calculate cluster variance values, hence we referred the value used in a similar study conducted by Holt et al. (28) and the rule-of-thumb suggested by others (29, 30).

Sampling was undertaken proportionate to the number of farms in each region, with farms selected using simple random selection. All cattle aged over 6 months on each selected farm were then selected for sample collection. The final sample included data from 87 and 127 randomly selected farms from Maekel and Debub regions, respectively. The mean and median herd size of the sampled farms was 15.1 and 9 head of cattle, respectively (minimum 5, maximum 158).

### 2.3. Data and sample collection

Some days prior to the day of blood and data collection, representatives of the household (farmers), the administrators of the selected zones, sub-zones, village administrators and village elders, were communicated through the respective regional veterinary officers, and well informed about the project. On the eve of the sampling date, owners of the selected farm were reminded again to stay at their farm and requested to collaborate with the survey team as they completed data collection. On the visiting day, members of the research team explained the purpose of the study to prospective participants and obtained written consent.

A questionnaire was designed to assess brucellosis-associated risk factors in the selected dairy farms though interviewing dairy farmers, family members or farm workers. The questionnaire content was focused on socio-demography, management/husbandry system, and individual animals risk factor data. For the socio-demography part, data on age, sex, educational level, relationship to the farm, and years of work experience of the respondent were recorded. For the management/husbandry questions, data on herd size, barn size, management system, source of water, restocking practices, history of reproductive disorders in animals, contact with other herds, presence of calving pens, hygienic status of the farms, ventilation, methods of disposal of waste and aborted materials were recorded. Concerning individual animal risk factors, data on age, sex, breed, parity, reproductive status, reproductive disorders (in the past 12 months), and origin were recorded for each individual animal. The questionnaire was pre-tested in 10 households before beginning the survey and necessary adjustments were made to improve clarity given the farming context.

Approximately 10 mL of blood was collected from the jugular/coccygeal vein of each animal using a plain vacutainer tube and double-ended needle. Each animal sample was labelled using a unique code given to the specific animal in the herd/farm. The sample tubes were set tilted on a table in a shed for about 12–18 h at a room temperature to allow separation of the serum from the other clots while protected from direct sunlight. When the serum was clearly separated from the other clots, it was decanted to another serum tube and labelled with the same code. The sera were subsequently transported in an ice box to the National Animal and Plant Health Laboratory (NAPHL), Ministry of Agriculture, Asmara for processing.

The authorities in the respective study regions gave permission for the research to be conducted in the study areas. Informed written consent was obtained from participants following a detailed explanation of the study objectives. Participation was voluntary and participants were informed of their right to withdraw at any time. All information provided by respondents including any identifiable information was kept strictly confidential. At the time of visit, animals were handled following standard procedures to ensure animal welfare. Blood samples were taken by veterinary professionals while applying all recommended precautions and safety procedures.

### 2.4. Laboratory investigation

#### 2.4.1. Rose Bengal plate test

Serum samples were screened for brucellosis antibody using a commercial Rose Bengal Plate Test (RBPT) kit (Animal Health and Veterinary Laboratory Agency; Surry, United Kingdom) following the manufacturer’s directions. Briefly, undiluted serum samples and antigen were adjusted to room temperature (18–25°C). Subsequently, 30 μL of serum was mixed with an equal volume of antigen on a glass plate to produce a zone approximately 2 cm in diameter. Each sample was thoroughly mixed using a disposable stirring stick, ensuring it was spread over the full surface of the circle. The mixture plate was rotated manually for 4 min at ambient temperature. The results for each sample were recorded by strength of agglutination observed for each antigen. Negative and positive controls for *B. abortus* and *B. melitensis* were included at the beginning of each testing session.

#### 2.4.2. Competitive enzyme-linked immuno-sorbent assay

RBPT positive sera were subjected to confirmatory testing using a commercial c-ELISA kit (INGNASA: Adeva Dela Institucion Libre de Ensenanza; Madrid, Spain) according to manufacturer’s directions. Briefly, 20 μL of each test serum was added to a 96-well plate pre-coated with *Brucella* spp. lipopolysaccharide (LPS) antigen and 100 μL of control solution added to duplicate wells. Plates were sealed and incubated at 20–25°C for 1 h. Consequently, the plate was washed 4 times, 100 μL conjugate added to each well and incubated at 20–25°C for 1 h. Again, the plate was washed 4 times, 100 μL substrate added to each well and incubated at 20–25°C for 10 min. After adding 100 μL stop solution to each well, optical density (OD) of each well read at 450 nm using plate reader machine, within 5 min. Results were considered valid if the OD in the negative control wells (NC) were >1 and the OD in the positive control wells (PC) were <0.35. Percentage of inhibition (PI) of each sample was calculated as: PI = 100 × [1 − (OD sample/OD negative control)]. Samples with PI >=40% were considered positive for *Brucella* antibody while samples with PI <40% were considered negative.

### 2.5. Data management and analysis

Questionnaire data and laboratory results were recorded and coded using Microsoft Excel. Serum samples that tested positive for both RBPT and c-ELISA were considered sero-positive for brucellosis. A herd/farm with one or more sero-positive animal was defined as a positive herd. Apparent prevalence (individual animal) was calculated as number of positive animals divided by the total number of animals tested. True prevalence was estimated in Epitools^2^ using the combined sensitivity and specificity above (83 and 100%). Similarly, apparent herd-level prevalence was computed as number of sero-positive herds divided by the total number of herds tested. Using Epitools^3^ we calculated that the herd sensitivity and specificity for testing in a large population, assuming a design prevalence of 7% and combined test sensitivity and specificity as above, was 100 and 100%, respectively. Therefore, apparent and true prevalence at herd level was deemed to be equivalent.

Descriptive (frequencies/percentages) and univariable logistic regression analysis was performed in SPSS version 23. Potential risk factors were investigated at both individual animal and herd levels, with herd-level variables further categorized into factors associated with herd-to-herd brucellosis transmission and factors associated with persistence of brucellosis in infected farms as done by Radostits et al. (4). Putative risk factors were initially screened for significant associations using Chi-square or Fisher’s exact test (discrete variables) or independent sample *t*-tests (continuous variables). Univariable logistic regression analysis was also applied to determine strength of association.

Variables with *p* < 0.25 were considered for inclusion in multivariable models. Before introducing variables into the model, candidate variables were checked for collinearity (correlation). Phi and Cramer’s V were used to check correlation between discrete variables, while Pearson correlation test was used to check for correlation between continuous variables. Combinations of variables scoring above 0.5 in Cramer’s V or Pearson test were denoted collinear. Where collinearity was observed between variables, the variable included in the multivariate model was selected following consideration of the level of statistical significance as well as biological plausibility. Collinearity was observed between the continuous variables of herd size (inclusive of male and female animals of all ages) and the number of cows. Thus, the number of cows was chosen for further analysis considering the significant role of cows in carrying and transmission of brucellosis in the herd. Similarly, correlation was observed between abortion (presence/absence) and abortion incidence; abortion (presence/absence) was chosen for further analysis.

Separate multi-variable models were built to investigate risk factors for brucellosis at individual animal (model 1) and herd level (model 2). For the herd level model, intermediate models exploring factors associated with inter-herd transmission and persistence of brucellosis on the farm were developed however only the final model combining both risk categories is reported. Given the hierarchical structure of the data, multivariable models were fitted using mixed effect models in R 4.2.3 software using the lme4 package with herd and village fitted as a random effects for the individual animal and herd level model, respectively. Models were developed using a forward selection and manual elimination process. Model building was started by introducing the variables with the lowest *p*-value. Subsequently, *p* < 0.05 was used as a cut-off value for eliminating variables from the models. Potential confounders were screened at every step of introducing a new variable to the existing model. Variables with 20% or more change in the odds ratio (OR) of another variable was used as the criterion for identifying confounders, however no confounder was identified in our model building process. *R*^2^ statistics were used to assess the goodness-of-fit for each model and explanatory power of each model was tested using Akaike’s Information Criteria (AIC).

## 3. Results

### 3.1. Respondent characteristics

Overall, 214 farms were included in this study, with one person (owner/worker) from each farm responding to the questionnaire. The majority (83.6%) of participants were males, nearly three quarters (74.3%) of whom were aged more than 30 years. Most (61.5%) had attended junior and secondary school. A majority (67.1%) of the sampled dairy farms were managed by family members. Most respondents had significant dairy farming experience with 43.5 and 35.5% having 6–15 years’ experience and greater than 15 years’ experience, respectively.

### 3.2. Individual- and herd-level prevalence of brucellosis

**Figure 1:**
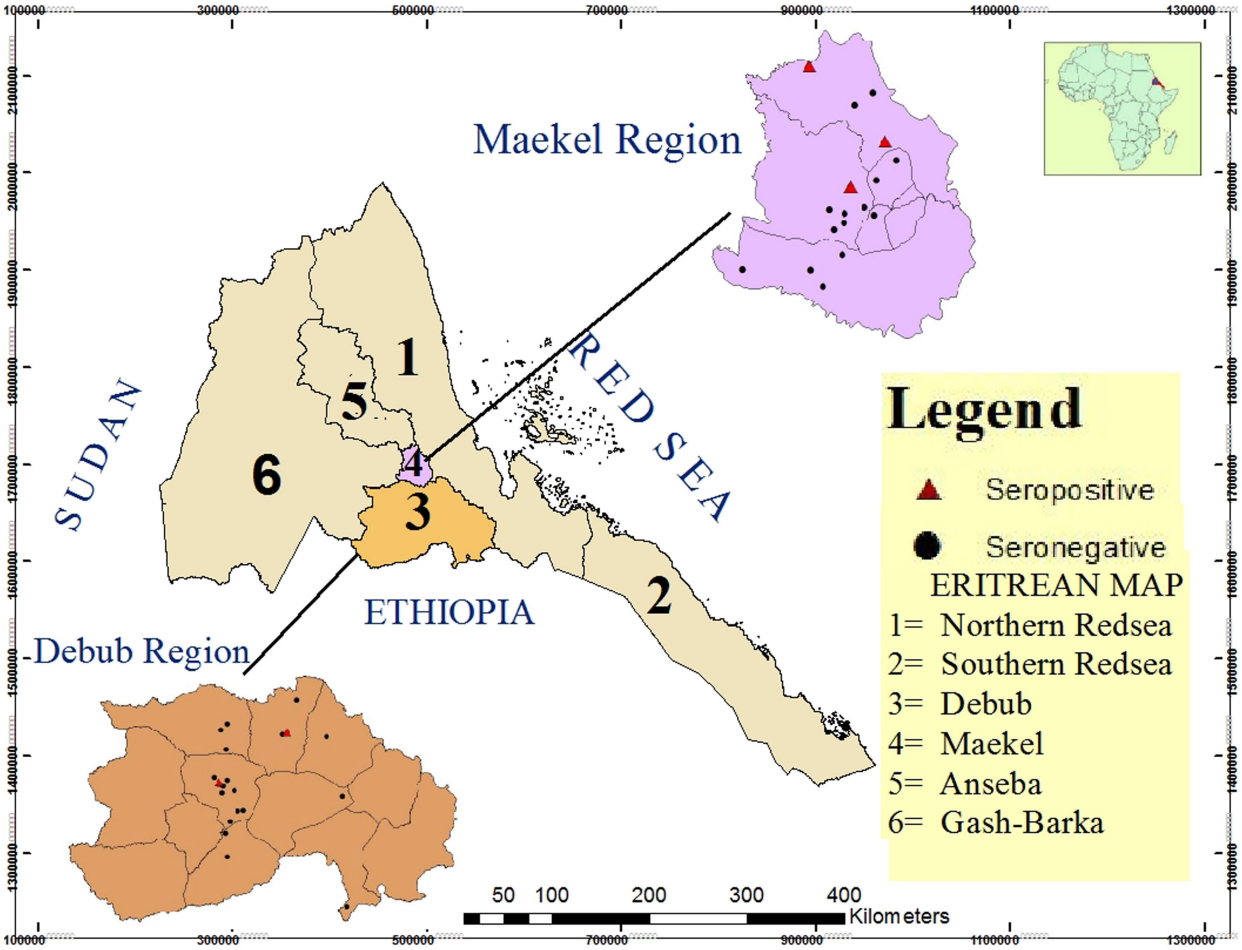
Map of Eritrea showing the location of the two study regions (Maekel and Debub) and specific location of sampled villages (*n* = 41) in the study sub-regions. Sampled villages with *Brucella* seropositive animals are represented as red triangles (*n* = 5) while sampled villages with no seropositive animals are indicated with black dots (*n* = 36). Source of map: Projection Datum 1984 UTM Zone 37 N.

A total of 2,740 dairy cattle from 214 herds in Maekel and Debub regions were tested for *Brucella* antibodies. The sampled animals came from 41 villages in the study sub-regions (Figure 1). In total, 34 animals tested positive by RBPT, 29 of which were confirmed positive by c-ELISA. Thus, the overall apparent prevalence in individual animals was 1.1% (95% CI: 0.7, 1.5%). Taking imperfect tests into account, the overall true prevalence in individual animals was estimated to be 1.3% (95% CI: 0.9, 1.8%). Animals that tested positive by RBPT came from 16 herds, all but one of which was confirmed positive on c-ELISA. Thus, the apparent and estimated true herd-level prevalence was 7.0% (95% CI: 4.3, 11.2%). The seroprevalence of brucellosis in small (5–10 heads), medium (11–50) and large (>50) sized herds was 1.3% (2/158), 16.7% (8/48), and 62.5% (5/8), respectively. Since there was no history of vaccination against brucellosis in dairy cattle in the study regions, natural infection was considered the cause of sero-positive findings.

Table 1 shows the results of *Brucella* testing by region and sub-region. In Maekel, 1.6 and 9.2% individual and herd-level prevalence was observed, respectively, with all positive detections contained within two sub-regions (Berik and Serejeka). In Debub, 0.6 and 5.5% individual- and herd-level prevalence was observed, respectively, with all positive detections in Dekemhare and Mendefera sub-regions.

**Table 1:** Apparent prevalence of brucellosis in dairy cattle in Maekel and Debub regions of Eritrea, stratified by sub-region (2022).

Region	Sub-region	Number of animals tested	RBPT positive, *n* (%)	c-ELISA positive, *n* (%)	95% CI	
					Lower	Upper
Maekel	Berik	805	15 (1.9)	15 (1.9)	1.0	2.9
	Serejeka	199	6 (3.0)	6 (3.0)	1.0	5.5
	Galanefhi	121	0	0		
	Asmara	216	0	0		
Sub-total		1,341	21 (1.6)	21 (1.6)	0.9	2.3
Debub	Adiquala	18	0	0		
	Dekemhare	527	10 (1.9)	6 (1.1)	0.4	2.1
	Dubarwa	220	0	0		
	Emnihaili	63	0	0		
	Mendeferra	521	3 (0.6)	2 (0.4)	0	1.0
	Segeneyti	50	0	0		
Sub-total		1,399	13 (0.9)	8 (0.6)	0.2	1.0
Grand total		2,740	34 (1.2)	29 (1.1)	0.7	1.5

### 3.3. Individual-level factors associated with brucellosis

Table 2 shows the prevalence of brucellosis stratified by individual animal characteristics. According to physiological status, the highest prevalence (2.2%) was found in non-pregnant lactating cows followed by dry cows (1.7%). Female animals had a higher rate of sero-positivity (1.1%) compared to male animals (0.5%). Most sampled animals were either Holstein Friesian (HF) (89.7%) or cross-breed (a mix of HF and local breeds) (7.9%), which had a 1.1 and 0.5% sero-prevalence, respectively. All local breed animals (*n* = 65) tested negative in both tests. Most (90.6%) animals were born or raised within the herd, of which 1% were sero-positive. Animals that were brought in (introduced) to the herds had a 1.6% positivity rate. Parity number ranged from 1 to 13 calves per a cow with a median of 3 and most observed frequency of 2 calves. No significant difference was observed in sero-positivity between cows having 1–3 calves (1.4%) compared to those that had ≥4 calves (1.8%). In the last 12 months, 35, 24, and 11 cows were reported to have experienced abortion, repeated breeding and retained placenta disorders, respectively. However, only one animal with abortion history tested sero-positive for brucellosis (1/35; 2.9%) and none of the animals with other disorders tested sero-positive. Of all individual risk factors associated with brucellosis, only region and physiological status had a *p*-value <0.25 and were considered for multivariable regression.

**Table 2:** Individual-level factors associated with brucellosis in dairy cattle in Maekel and Debub regions of Eritrea.

Variable	Category	Number of animals tested	c-ELISA positive, *n* (%)	*p*-value	Odds ratio	95% CI	
						Lower	Upper
Physiological status	Calf^a^	637	5 (0.8)	0.014	Ref.		
	Non pregnant lactating cows^b^	741	16 (2.2)		2.790	1.016	7.658
	Pregnant lactating cows^c^	560	3 (0.5)		0.681	0.162	2.862
	Pregnant heifer^d^	281	0		ND		
	Dry cows^e^	231	4 (1.7)		2.227	0.593	8.367
	Non pregnant heifer^f^	209	1 (0.5)		0.608	0.071	5.231
	Bull^g^	81	0		ND		
Sex	Male	191	1 (0.5)	0.758	Ref.		
	Female	2,549	28 (1.1)		2.088	0.283	15.431
Breed	Holstein Frisian	2,459	28 (1.1)	0.452	2.488	0.337	18.372
	Cross-breed	281	1 (0.5)		Ref		
	Local	65	0		ND		
Origin	Born/raised in the herd	2,482	25 (1.0)	0.348	Ref		
	Recently introduced to the herd	258	3 (1.6)		1.548	0.534	4.482
Region	Maekel	1,341	21 (1.6)	0.014	2.766	1.221	6.267
	Debub	1,399	8 (0.6)		Ref		
History of abortion in the last 12 months	Yes	35	1 (2.9)	0.415	1.972	0.258	15.054
	No	1,497	22 (1.5)		Ref		
	Total	1,532	23				
History of retained placenta in the last 12 months	Yes	11	0	ND			
	No	1,521	23 (1.5)				
	Total	1,532	23				
History of repeat breeding in the last 12 months	Yes	24	0	ND			
	No	1999	24 (1.2)				
	Total	2023	24				

For all variables except reproductive disorders the denominator for the prevalence calculation is the total number of sampled animals (*n* = 2,740). For reproductive disorders, the denominator is stated in the table and reflects the number of cows that were reproductively active. *p*-values were derived from chi-square or Fisher’s exact test. Univariable logistic regression was used to derive the crude odds ratio (OR) and associated confidence interval (CI). Comparisons between categories with zero cases were not done (ND).

a Animal aged <2 years.

b Cows with ≥1 parturitions and actively lactating.

c Cows with ≥1 parturitions, pregnant, and actively lactating.

d Heifer aged ≥2 years, conceived for their first pregnancy.

e Cows with ≥1 parturitions and not lactating.

f Heifer aged >=2 years, not conceived for their first pregnancy.

g Bull aged >=2 years.

### 3.4. Herd-level factors associated with brucellosis

The prevalence of brucellosis stratified by herd-level characteristics is shown in Table 3 (categorical variables) and Table 4 (continuous variables). In terms of factors associated with inter-herd transmission, farms that allowed animals to graze outdoors had a lower prevalence compared to those that always kept animals indoors (9.4% vs. 2.7%; OR = 0.266, *p* = 0.09). Farms that use natural mating had a lower prevalence than those practiced artificial insemination (AI) or use both AI and natural mating (4.8% vs. 10.0%; OR = 0.458, *p* = 0.178). Farms that used non-farm bulls for breeding had a reduced prevalence compared to farms that did not use non-farm bulls (3.8% vs. 11.8%; OR = 0.295, *p* = 0.057). Notably, only 2.3% (5/214) farms tested new animals for brucellosis before introducing them into their herds.

**Table 3:** Herd-level factors (categorical variables) associated with brucellosis in dairy cattle in Maekel and Debub regions of Eritrea.

Variable	Category	Number of farms	c-ELISA positive, *n* (%)	*p*-value	Odds ratio	95% CI	
						Lower	Upper
Factors associated with inter-herd transmission							
Source of drinking water	Tap water/farm well inside the farm	82	8 (9.8)	0.272	1.931	0.673	5.541
	Shared/communal source outside the farm	132	7 (5.3)		Ref		
Proximity of neighboring dairy farms (meter)	≤10	50	5 (10)	0.464	2.306	0.589	9.018
	11–100	77	6 (7.8)		1.754	0.476	6.641
	>100	87	4 (4.6)		Ref		
Management system	Animals always kept indoors	139	13 (9.4)	0.092	Ref.		
	Animals allowed to graze outdoors	75	2 (2.7)		0.266	0.058	1.210
Replacement of stock animals by purchase	Yes	66	5 (7.6)	0.780	1.131	0.371	3.450
	No	148	10 (6.8)		Ref		
Test animals for brucellosis before purchase	Yes	5	1 (20)	0.307	3.482	0.364	33.283
	No	209	14 (6.7)		Ref		
Breeding method	Natural mating only	124	6 (4.8)	0.178	0.458	0.157	1.335
	Artificial insemination/both	90	9 (10.0)		Ref		
Use of non-farm bulls for breeding	Yes	105	4 (3.8)	0.057	0.295	0.091	0.962
	No	93	11 (11.8)		Ref		
Factors associated with persistence of brucellosis within a herd							
Presence of other domestic animals in the farm	Yes	191	11 (5.8)	0.062	0.290	0.084	1.001
	No	23	4 (17.4)		Ref		
Sheep on the farm	Yes	35	1 (2.9)	0.475	0.347	0.044	2.725
	No	179	14 (7.8)		Ref		
Goat on the farm	Yes	14	2 (14.3)	0.256	2.397	0.485	11.863
	No	200	13 (6.5)		Ref		
Horse on the farm	Yes	57	5 (8.8)	0.551	1.413	0.462	4.328
	No	157	10 (6.4)		Ref		
Dog on the farm	Yes	155	6 (3.9)	0.006	0.224	0.076	0.660
	No	59	9 (15.3)		Ref		
Donkey on the farm	Yes	100	3 (3.0)	0.035	0.263	0.072	0.960
	No	114	12 (10.5)		Ref		
Housing type	Stanchion system	155	15 (9.7)	ND			
	Traditional	58	0				
Farm floor	Concrete	172	15 (8.7)	ND			
	Non-concrete	40	0				
Aborted foetus/foetal membrane disposal method	Bury/burn	36	0	ND			
	Feed to dogs	131	11 (8.4)				
	Leave in place	9	0				
	Dispose into rubbish dump	38	4 (10.5)				
Separate calving pen	Yes	19	0	ND			
	No	195	15				
Presence of bull/s on the farm	Yes	102	13 (12.7)	0.002	8.034	1.766	36.538
	No	112	2 (1.8)		Ref		
Abortion observed in the herd on the past 12 months	Yes	29	5 (17.2)	0.036	3.646	1.148	11.574
	No	185	10 (5.4)		Ref		
Retained placenta observed in the herd in the past 12 months	Yes	15	1 (6.7)	1.000	0.944	0.116	7.710
	No	199	14 (7.0)		Ref		
Repeat breeding observed in the herd in the past 12 months	Yes	46	2 (4.3)	0.534	0.542	0.118	2.493
	No	168	13 (7.7)		Ref		

Univariable logistic regression was used to derive the crude odds ratio (OR) and associated confidence interval (CI). *p*-values were derived from chi-square or Fisher’s exact test. Comparisons between categories with zero cases were not done (ND).

**Table 4:** Herd-level factors (continuous variables) associated with brucellosis in dairy cattle in Maekel and Debub regions of Eritrea.

Variable	c-ELISA result	Mean	*p*-value	Odds ratio	95% CI	
					Lower	Upper
Herd size	Positive	49.53	<0.001	1.066	1.037	1.095
	Negative	12.50				
Number of cows	Positive	26.53	<0.001	1.139	1.075	1.208
	Negative	6.15				
Proximity of neighboring farms (meter)	Positive	107.86	0.413	0.998	0.995	1.002
	Negative	150.61				
Number of abortions recorded in the herd in the last 12 months	Positive	0.91	0.059	1.65	0.950	2.871
	Negative	0.39				

*p*-values were derived from independent sample *t* tests.

In terms of factors associated with persistence of brucellosis in infected farms, presence of bull (OR = 8.034; *p* = 0.002), history of abortion on the farm in the last 12 months (OR = 3.646; *p* = 0.036), herd size (OR = 1.066; *p* < 0.001) and number of cows on the farm (OR = 1.139; *p* < 0.001) were significantly associated with brucellosis sero-positivity. Presence of dogs (OR = 0.224; *p* = 0.006) and donkeys (OR = 0.263; *p* = 0.035) on the farm was negatively associated with brucellosis, while presence of goats was associated with higher rates of sero-positivity compared to farms without these animals (14.3% vs. 6.5%, *p* = 0.256). Improper disposal of aborted foetuses/placentas by feeding to dogs or disposing in rubbish dump was practiced by the majority of farms (169/214) and associated with the highest sero-positivity (8.4 and 10%, respectively). In contrast, farms that practiced proper disposal by burying or burning had no sero-positive animals. The majority (81%) of the farms had no calving pens; farms that did own calving pens had no sero-positive animals.

### 3.5. Multivariable analysis

Table 5 shows the final multivariable models describing individual- and farm-level risk factors for brucellosis. Both physiological status and study region were considered for inclusion in the individual-level multivariable model, however only physiological status was retained (model 1). Thus, after accounting for the hierarchical nature of the data, non-pregnant lactating cows were 3.4 (*p* = 0.042) times more likely to be seropositive. A number of variables potentially related with inter-herd transmission (management system, breeding method and use of non-farm bulls) and persistence of brucellosis on the farm (number of cows, presence of dog, presence of bull/s on the farm, abortion in past 12 months) were considered for inclusion in intermediate herd-level multivariable models, however only history of abortion and number of cows were retained in the final combined model (model 2). Thus, in the adjusted analysis, for every cow that is present on a farm, the risk of *Brucella* sero-positivity on the farm increases by approximately 14% (*p* < 0.001). Further, the odds of brucellosis were approximately 6 times higher in farms with history of abortion compared to farms that did not have such history (*p* = 0.026).

**Table 5:** Final multivariable models describing individual (model 1) and herd level (model 2) risk factors for brucellosis in dairy cattle in Maekel and Debub regions of Eritrea.

Variable	Category	*p*-value	aOR	CI at 95%	
				Lower	Upper
Model 1: Individual-level risk factors for brucellosis					
Physiological status	Calf	Ref			
	Non-pregnant heifer	0.647	1.76	0.16	19.68
	Non-pregnant lactating	0.042	3.35	1.04	10.80
	Pregnant lactating	0.981	0.98	0.20	4.74
	Dry cow	0.164	2.88	0.65	12.81
Model 2: Herd-level risk factors for brucellosis					
Abortion observed in the herd in the past 12 months	Yes	0.026	5.71	1.24	26.38
Number of cows	NA	<0.001	1.14	1.07	1.22

Adjusted odds ratios (aOR) were derived from multivariable mixed effect models with herd and village fitted as a random effects in model 1 and 2, respectively. *p*-values were derived from the test of significance for each coefficient in the model.

## 4. Discussion

This study aimed to provide updated findings on the prevalence of and risk factors for brucellosis in dairy animals in Eritrea. In this study, the overall individual- (1.1%) and herd-level (7.0%) sero-prevalence was low. This result was lower than previously reported by Omer et al. [8.2 and 35.9% individual and herd-level prevalence in Asmara city, respectively, using RBPT as screening and CFT as confirmatory test; (17)] and Scacchia et al. [2.8% individual-level prevalence across 5 regions of Eritrea using RBPT as screening and CFT as confirmatory test; (19)], and relatively similar to reports from Samson [individual and herd level sero-prevalence of around 3 and 2%, respectively, in Berik sub-region of Maekel region using RBPT and milk ring test; (20)]. We note that these researchers sampled reproductively mature animals which may explain the higher prevalence in these studies. However, a sub-analysis of apparent prevalence in mature animals in our study confirms that prevalence is indeed lower than previously reported (24/2103; 1.1%). The variation in reported prevalence may be due to differences in testing modalities, sampling design and other factors such as husbandry practices. The low prevalence could also be associated with the on-going ‘dairy cattle movement control’ including ‘test and slaughter’ measure applied by the Regulatory Services Department (RSD), Ministry of Agriculture, Eritrea following the Legal Notice No.113/2006 published in the year 2006 (30). We also note that the great majority (90.6%) of study animals were born or raised within the herd, indicating that only a small proportion of farms were at risk of introducing brucellosis through purchased animals. This practice may also have contributed to the low recorded prevalence in the study areas.

The low prevalence reported in this study is comparable to estimates reported by some authors in neighboring Ethiopia, where prevalence was 1.5% in Addis Ababa dairy farms (31), 1.4% in agro-pastoral areas in Jijiga zone Somali region (32) and 1.7% in cattle from milk cooperatives in Arsi zone Oromia region (33). Other authors have reported lower prevalence rate. For instance: 0.06 and 0.8% individual and herd level seroprevalence in Addis Ababa dairy farms, respectively (34); 0.2% in cattle in Debrebirhan and Ambo towns in Amhara and Oromia regions of Ethiopia, respectively (35) and 0.26% in cattle in Yemen (36). On the other hand, a much higher sero-prevalence has been reported in other areas such as 50% in Borena zone of Oromia, Ethiopia (37) and 31.1% in Khartoum, Sudan (38). This marked variation in sero-prevalence might be due to various reasons including differences in study area (highland, lowland; location, size of covered area), study population (exotic, indigenous breed type), population dynamics (herd size, density, proportion of various breeds), biological features of the causative organisms (*B. abortus*, *B. melitensis*), management practices (good, moderate, poor), and laboratory test methods applied (RBPT, ELISA, CFT, MRT, and SAT), as explained by several authors (39–41).

Despite the low prevalence, when stratified by the two study regions, our univariable analysis showed a statistically higher individual-level prevalence in Maekel than in Debub region. While this finding was not confirmed in multivariable analysis it is perhaps notable that Scacchia et al. (18) reported a similar observation between the two regions (though did not analyze its significance). If confirmed, we speculate that the observed difference could be linked to the higher number of large herds in Maekel region as well as a longer endemic history of brucellosis in Maekel compared to Debub.

Physiological status was the only risk factor retained in the final model describing individual-level risk factors for brucellosis, with non-pregnant lactating cows found to be significantly more likely than calves to be sero-positive. Brucellosis is considered a disease of adult animals since susceptibility increases after sexual maturity and pregnancy (42, 43). This happens because sex hormones and erythritol tend to increase in concentration with age and sexual maturity and can stimulate the growth and multiplication of *Brucella* organisms, (42, 44). On the other hand, younger animals are often resistant to *Brucella* infection although latent infections can occur (4).

Presence of a bull on the farm was strongly associated with persistence of brucellosis in univariable analysis but ultimately found to be non-significant in multivariable models. Sharing of bulls is a common practice in Eritrea (45). Bulls may increase the risk of infection as they have frequent contact with other herds during the breeding period (46). According to Radostits et al. (4) infected bulls are unlikely to transmit brucellosis by natural mating, but if its semen is used for artificial insemination (AI) the chance of spreading the disease is very great. AI was practiced by around 40% of farms in this study. Notably, though, all bulls tested in this study were sero-negative for brucellosis.

Increasing number of cows on a farm was significantly associated with brucellosis risk in herds in multivariable analysis in this study. Cows have an active reproductive cycle (pregnancy, parturition, or lactating) which is favorable for maintaining *Brucella* in the environment. Hence, the higher the number of cows on the farm, the greater the opportunity for persistent transmission between animals. Large herd size is a well-recognized risk factor for *Brucella* infection (47). This is related to the fact that large sized herds are usually associated with intensive management practices that make control more difficult, increasing the potential for exposure to infectious excretions and fomites (17, 48–50).

In multivariable models, the risk of brucellosis in herds was also associated with the history of abortion on a farm. This association can be expected since brucellosis usually manifests through abortion (51–53). Abortion facilitates the release of large numbers of *Brucella* organisms which can contaminate the environment and subsequently be ingested by susceptible animals (54). Farms can experience huge economic losses due to brucellosis as a result of abortion, calf death, decreased milk production, permanent infertility, prolonged inter-calving period and culling of infected cows among the other problems (4, 5, 55).

Farming several species in the same herd is a high-risk practice for brucellosis transmission as broadly discussed by Coelho et al. (12). Keeping goats was associated with higher odds of persistence in this study, although this was non-significant in univariable analysis (and likely underpowered due to small numbers). Infected goats can excrete *Brucella* species for 2–3 months following abortion or normal parturition (56). Keeping of dogs and donkeys was associated with lower odds of persistence on the farm in univariable analysis, although neither were included in the final multivariable model. If anything, presence of dog in herds is likely to increase transmission within and between herds by dragging infected placentas, aborted fetuses, and other birth materials thereby contaminating the environment (57). Further, seroprevalence rates were highest in farms that disposed of aborted fetuses and placentas by feeding to dogs. Considering the biological nature and behavior of *Brucella* species and donkeys, the presence of donkeys in a herd is unlikely to reduce the risk of brucellosis.

This study also revealed that most farms had poor management practices at the time of the study which expose animals and humans to the risk of brucellosis. A high proportion (81%) of the sampled farms had no calving pens and it was notable that no sero-positive animals were detected on the 19 farms that did have calving pens. Isolation of post-parturient animals in maternity facilities such as calving pens reduces the risk of spread of brucellosis infection to the rest of the herd or flock (58). In addition, most (97.7%) farms did not test newly purchased animals for brucellosis before introducing them into their herds, increasing the chances of introducing brucellosis into naïve herds. Improper disposal of aborted fetuses/placentas by feeding to dogs or disposing into rubbish dumps was also observed in infected farms. Again, it is notable that no sero-positive animals were detected in farms that followed proper disposal practices. Therefore, raising awareness of dairy farmers on good management practices is urgently needed to tackle the risk of spreading brucellosis and assuring public health safety.

There are a number of limitations to this study. Due to resource constraints, the current study targeted only farms owing more than four animals. As a result, a great proportion of dairy herds in the study were not included. For similar reasons, the study was localized to two regions out of a total of five regions where dairy cattle are raised. Some of the contrary results revealed in our study need more investigations for further explanation.

## 5. Conclusion

Findings of this study have confirmed a low prevalence of brucellosis in dairy cattle in Maekel and Debub regions, Eritrea. The individual and herd level sero-prevalence of brucellosis in the two study regions was low and lower than previously reported. History of abortion, number of cows in a herd, physiological status were independently and significantly association with brucellosis sero-positivity, hence have been recognized as the most important risk factors for brucellosis transmission and persistence in the study area. According to the present study findings, it is feasible to control the disease in the studied area following strict test-and slaughter policy and procedures.

### 5.1. Recommendations

In the absence of strict controlling measures, brucellosis can spread widely between animals and herds and to humans. Therefore, animal movement control, good farming practices, sanitary and biosecurity measures, training, and awareness raising programs on brucellosis are recommended to be implemented in the study areas to limit transmission.

## Data availability statement

The raw data supporting the conclusions of this article will be made available by the authors, without undue reservation.

## Ethics statement

The studies involving human participants were reviewed and approved by the ethical committees of the Ministry of Health in Eritrea, the International Livestock Research Institute IREC (IREC2021-01) and the University of Liverpool REC (reference 9,835). The patients/participants provided their written informed consent to participate in this study. The animal study was reviewed and approved by the International Livestock Research Institute IACUC (IACUC2021-01). Written informed consent was obtained from the owners for the participation of their animals in this study.

## Author contributions

The research protocol was designed and drafted by GE then revised with input from by SM, MG, BM, GM, and YG. GE, BM, and TO participated in sample/data collection and laboratory analysis. Data analysis was performed by GE and BM. GE, BM, MG, TO, YG, SM, and GM contributed to interpretation of the results and revision of the manuscript. All authors contributed to the article and approved the submitted version.

## Funding

This project was partially supported by the Global Challenges Research Fund (GCRF) One Health Regional Network for the Horn of Africa (HORN) Project, from UK Research and Innovation (UKRI) and Biotechnology and Biological Sciences Research Council (BBSRC) (project number BB/P027954/1). Laboratory consumables were funded and supplied by the Ministry of Agriculture, State of Eritrea.

## Conflict of interest

The authors declare that the research was conducted in the absence of any commercial or financial relationships that could be construed as a potential conflict of interest.

## Publisher’s note

All claims expressed in this article are solely those of the authors and do not necessarily represent those of their affiliated organizations, or those of the publisher, the editors and the reviewers. Any product that may be evaluated in this article, or claim that may be made by its manufacturer, is not guaranteed or endorsed by the publisher.

## Footnotes

^1^ https://epitools.ausvet.com.au/prevalencess

^2^ https://epitools.ausvet.com.au/trueprevalence

^3^ https://epitools.ausvet.com.au/herdsensone
